# Prioritization and Severe Primary Graft Dysfunction after Lung Transplantation

**DOI:** 10.1111/ctr.70711

**Published:** 2026-10-14

**Authors:** Flávio Pola dos Reis, Alexandre Mestre Tejo, Samuel Lucas dos Santos, Flavia Fiuza de Andrade Vale, Victor Arayama Cruz, Luis Gustavo Abdalla, Silvia Vidal Campos, Lucas Matos Fernandes, Paulo Manuel Pêgo‐Fernandes

**Affiliations:** ^1^ Departamento De Cirurgia Torácica Instituto Do Coração do Hospital das Clínicas da Faculdade de Medicina da Universidade de São Paulo São Paulo Brazil; ^2^ Infectious Diseases and Tropical Medicine Department Hospital Das Clínicas da Faculdade de Medicina da Universidade de São Paulo São Paulo Brazil; ^3^ Departamento De Pneumologia Instituto Do Coração do Hospital das Clínicas da Faculdade de Medicina da Universidade de São Paulo São Paulo Brazil

**Keywords:** Extracorporeal membrane oxygenation (ECMO), Ischemia–reperfusion injury, Lung transplantation, Primary graft dysfunction, Transplant prioritization

## Abstract

**Background:**

Primary graft dysfunction (PGD) is the leading cause of early morbidity and mortality after lung transplantation (LTx). Critically ill candidates may receive priority on the transplant waiting list; however, the relationship between prioritization and the development of severe PGD remains unclear.

**Methods:**

We performed a retrospective analysis of a prospectively maintained cohort including all consecutive LTx performed at a tertiary center between January 2016 and June 2025. Recipients were categorized according to prioritization status. Severe PGD was defined as grade 3 PGD according to the International Society for Heart and Lung Transplantation criteria. Clinical characteristics and outcomes were compared between prioritized and non‐prioritized recipients. Logistic regression was used to identify predictors of severe PGD, and survival was evaluated using Kaplan–Meier analysis.

**Results:**

Among 310 LTx recipients, 74 (23.8%) were prioritized. They were younger, had a higher prevalence of pulmonary hypertension, and were more frequently hospitalized before transplantation, particularly in the intensive care unit. Severe PGD occurred more often among prioritized recipients (24% vs 7%). However, prioritization was not independently associated with PGD after adjustment. Pre‐transplant extracorporeal membrane oxygenation support emerged as the strongest independent predictor of severe PGD. Thirty‐day mortality was similar between groups, whereas prioritized recipients had higher 90‐day and 1‐year mortality.

**Conclusions:**

Prioritized LTx recipients have greater pre‐transplant clinical severity, higher crude rates of severe PGD, and worse short‐term survival. However, prioritization itself was not independently associated with PGD, suggesting that underlying disease severity and need for advanced support are the main drivers of post‐transplant outcomes.

**Abbreviations**
ECMO: Extracorporeal Membrane Oxygenation
ICU: Intensive Care Unit
ISHLT: International Society for Heart and Lung Transplantation
LAS: Lung Allocation Score
LTx: Lung Transplantation
PGD: Primary graft dysfunction

## 1 Introduction

Lung transplantation (LTx) is an established therapy for selected patients with advanced pulmonary diseases, providing substantial survival and quality‐of‐life benefits. Advances in pretransplant therapies, bridging strategies, perioperative management, and post‐transplant immunosuppression have expanded the pool of transplant candidates while increasing the complexity of recipient selection. Most programs follow the International Society for Heart and Lung Transplantation (ISHLT) consensus recommendations to guide candidate evaluation, balancing disease severity, comorbidities, and expected survival benefit when determining listing strategies [1].

In this evolving landscape, patients experiencing acute pulmonary deterioration, previously considered unsuitable for transplantation, are increasingly evaluated under urgent or prioritized listing pathways when the anticipated survival benefit outweighs operative risk. Several studies suggest that long‐term survival among prioritized recipients may approximate that of standard‐list recipients [2, 3]. However, whether this apparent equivalence in long‐term outcomes conceals differences in early post‐transplant morbidity remains insufficiently explored.

Amid these outcomes, primary graft dysfunction (PGD) remains the leading cause of morbidity and mortality in the early postoperative period and represents the clinical manifestation of ischemia–reperfusion injury [4]. Its development reflects a complex interaction among recipient characteristics, donor variables, and perioperative management, amplified by inflammatory and immunological mechanisms [5]. Recipient factors such as pulmonary arterial hypertension, idiopathic pulmonary fibrosis, sarcoidosis, and right ventricular dysfunction have been consistently associated with increased PGD risk [6, 7]. Donor factors, including advanced age, female sex, and smoking history [7, 8], and operative variables such as extracorporeal membrane oxygenation (ECMO) and prolonged ischemic time also contribute substantially [7, 9].

Recipients listed under prioritization pathways frequently present with many of these risk factors at baseline. Although previous reports have addressed survival among prioritized recipients [2, 3], limited data directly evaluate the incidence and severity of PGD in this population compared with non‐prioritized recipients. Understanding whether prioritization status independently increases the risk of severe PGD and whether PGD may mediate early mortality in this subgroup is essential for refining allocation strategies and optimizing perioperative management.

Therefore, the objective of this study was to characterize prioritized lung transplant recipients, compare the incidence of severe PGD between prioritized and non‐prioritized recipients, identify factors associated with severe PGD, and evaluate the impact of prioritization on short‐term survival after LTx.

## 2 Materials and Methods

### 2.1 Study Design and Population

We conducted a retrospective analysis of a prospectively maintained cohort including all consecutive lung transplantations performed at the Heart Institute (InCor), Hospital das Clinicas (University of São Paulo, Brazil), between January 2016 and June 2025. All patients undergoing LTx were eligible regardless of age. The study was approved by the local Institutional Ethics Committee (CAAE: 39990120.8.0000.0068).

### 2.2 Allocation and Prioritization Policy

During the study period, lung allocation in Brazil was primarily based on chronological waiting time within state‐based transplant systems coordinated by the State Transplant Centers under the broader framework of the Brazilian National Transplant System. Unlike LAS‐based allocation systems, the Brazilian model did not use a continuous severity score integrating predicted waitlist mortality and expected post‐transplant survival. Instead, prioritization was granted as a binary urgency status after individual review by the State Transplant Center Technical Chamber. Formal prioritization was legally established for selected urgent scenarios, including acute graft dysfunction within 30 days after prior transplantation. In clinical practice, candidates with rapid clinical deterioration, refractory respiratory failure, need for invasive mechanical ventilation, or prolonged extracorporeal support could also be submitted for technical review and granted priority status. Recipients transplanted under this urgency‐based pathway were classified as prioritized. The main criteria considered for prioritization are summarized in Table 1 and compared with the LAS system.

**TABLE 1:** Comparison between LAS‐based allocation, the Brazilian prioritization system during the study period, and the new Brazilian allocation framework established in 2025.

Domain	LAS‐based allocation system	Brazilian prioritization system during the study period (2016–2025)	New Brazilian allocation framework (from 2025 onward)
**Allocation principle**	Continuous score‐based allocation integrating predicted waitlist mortality and expected post‐transplant survival benefit	Chronological waiting time was the primary allocation criterion, with prioritization granted as an exceptional binary status	Hybrid urgency‐based system with hierarchical prioritization criteria incorporated into the national allocation framework
**Type of urgency assessment**	Quantitative and continuous	Binary (prioritized vs. non‐prioritized)	Hierarchical severity‐based prioritization
**Candidate ranking**	Determined by calculated allocation score	Primarily determined by waiting time unless priority status was approved	Determined by predefined urgency categories combined with waiting‐list organization
**Main objective**	Balance urgency and expected post‐transplant benefit	Provide access to transplantation for selected candidates with rapid clinical deterioration	Standardize prioritization nationally and improve allocation according to clinical severity
**Organizational structure**	National or large regional allocation networks	State‐based transplant allocation coordinated by State Transplant Centers	Integrated within the National Transplant System (SNT), with national regulatory oversight and SIGA‐based management
**Priority assignment**	Automatically generated through score calculation	Granted after review by the State Transplant Center Technical Chamber	Defined according to nationally regulated severity levels
**Role of external review**	Limited; allocation follows predefined score calculation	Mandatory technical review before priority approval	Governed by nationally standardized allocation criteria and regulatory oversight
**Main drivers of urgency**	Waitlist mortality risk, diagnosis, oxygen requirement, functional status, hemodynamics, expected post‐transplant survival	Rapid clinical deterioration, respiratory failure, invasive mechanical ventilation, ECMO support, ICU admission, acute graft dysfunction, or exceptional circumstances	Severity levels formally include acute graft dysfunction, extracorporeal life support (ECLS), invasive mechanical ventilation, and waiting‐list status
**Role of ECMO**	Incorporated indirectly through variables affecting mortality prediction	Considered a major indication supporting urgency‐based prioritization	Explicitly recognized as a high‐priority severity criterion
**Role of invasive mechanical ventilation**	Incorporated indirectly through severity score variables	Considered individually during technical review	Explicitly incorporated into prioritization hierarchy
**National standardization**	High	Moderate; prioritization depended on case‐by‐case technical review	Higher degree of national standardization through federal regulation
**Relationship to the present study**	Not directly comparable	Represents the allocation model used during the study period (2016–2025)	Not applied to the present cohort, but reflects the current Brazilian allocation framework
**Implication for interpretation**	Results from LAS‐based cohorts may not be directly comparable	Findings reflect outcomes under a waiting‐time‐based system with urgency exceptions	Future Brazilian cohorts may show different recipient profiles and outcomes due to the new severity‐based prioritization structure

Abbreviations: ECLS, extracorporeal life support; ECMO, extracorporeal membrane oxygenation; LAS, Lung Allocation Score; SIGA, Sistema Informatizado de Gerenciamento do Acesso às Listas de Espera; SNT, Brazilian National Transplant System.

### 2.3 Definition and Assessment of Primary Graft Dysfunction

PGD was defined according to the ISHLT consensus criteria [11]. PGD grading was based on the PaO_2_/FiO_2_ ratio and radiographic evidence of pulmonary edema at 0, 24, 48, and 72 h after LTx. The grading system is illustrated in Table S1. In accordance with ISHLT recommendations, recipients requiring ECMO for hypoxemic respiratory failure with radiographic pulmonary edema were classified as PGD grade 3 regardless of oxygenation measurements.

### 2.4 Data Collection

Recipients, donors, intraoperative, and postoperative variables were collected from institutional databases and electronic medical records. Recipient variables included demographic characteristics, underlying diagnosis, pulmonary pressure status, kidney function, and pre‐transplant clinical condition. Pulmonary hypertension was defined as one of the following: a mean pulmonary artery pressure >25 mmHg on right heart catheterization; or as pulmonary artery systolic pressure >40 mmHg on pre‐transplant echocardiography; or as clinically significant pulmonary hypertension documented in the pre‐transplant evaluation based on echocardiographic and hemodynamic assessment. Pre‐transplant location was classified according to the patient's status at the transplant center immediately before transplantation. Patients classified as not hospitalized at the transplant center could have been hospitalized at an external referring institution before transfer. The database did not systematically capture the location or level of care at referring institutions. Donor variables included demographics, cause of death, oxygenation parameters, duration of mechanical ventilation, and time from brain‐death diagnosis to organ procurement. Operative variables included type of transplant, ischemic time, and use of intraoperative extracorporeal support. Extracorporeal life support (ECLS) was used as an umbrella term for intraoperative extracorporeal support, including VV‐ECMO, VA‐ECMO, and cardiopulmonary bypass. When the specific ECMO configuration was available, it was recorded accordingly; however, because configuration data were incomplete across the long study period, cases without reliable documentation were reported simply as ECMO to avoid misclassification. Intraoperative ECLS/ECMO was used for hemodynamic support in recipients with severe pulmonary hypertension, right ventricular dysfunction, or hemodynamic instability; for respiratory support in cases of intolerance to one‐lung ventilation, refractory hypoxemia, hypercapnia, or inadequate CO_2_ clearance; and, in selected cases, for technical reasons such as limited surgical exposure in recipients with a small thoracic cavity. Postoperative variables were analyzed as clinical outcomes associated with PGD.

### 2.5 Study Endpoints

The primary endpoint was PGD grade 3 at 72 h after transplantation. Secondary outcomes included mortality at 30 days, 90 days, and 1 year; post‐transplant ECMO requirement; duration of mechanical ventilation; intensive care unit (ICU) length of stay; and total hospital length of stay.

### 2.6 Statistical Analysis

Continuous variables are presented as mean or median, according to data distribution, and were compared using Student's *t* test or the Mann–Whitney *U* test. Categorical variables are reported as counts and percentages and were compared using the chi‐square test or Fisher's exact test.

The incidence of PGD grade 3 was evaluated at 0, 24, 48, and 72 h after transplantation. Logistic regression models estimated odds ratios (OR) with 95% confidence intervals (CI) comparing prioritized and non‐prioritized recipients. A model including prioritization status, time after transplantation, and their interaction was used to assess differences in PGD temporal evolution.

Variables clinically relevant or associated with PGD in univariable analyses were entered into a multivariable logistic regression model to identify independent predictors of severe PGD. Survival was assessed using Kaplan–Meier curves with log‐rank tests. Cox proportional hazards models estimated hazard ratios (HR) with 95% CI for factors associated with mortality. Survival was evaluated at 30 days, 90 days, and 1 year.

All analyses were performed using R software (R Foundation for Statistical Computing, Vienna, Austria). A two‐sided *p*‐value < 0.05 was considered statistically significant.

## 3 Results

A total of 310 lung transplant recipients were included. Among these, 74 (23.8%) underwent transplantation under prioritization, whereas 236 (76.2%) were transplanted according to the regular waiting list.

### 3.1 Recipient and Donor Characteristics

Baseline characteristics are summarized in Table 2. Prioritized recipients were younger than non‐prioritized recipients (36.6 vs 42.3 years; *p* = 0.007), while donor characteristics were similar between groups. Vascular and restrictive lung diseases were more frequent among prioritized recipients (12% vs 3% and 50% vs 37%, respectively; *p* < 0.001). Markers of disease severity were also more prevalent in the prioritized group, including pulmonary hypertension (48% vs 22%; *p* < 0.001) and higher pulmonary artery systolic pressure (66.5 vs 57.7 mmHg; *p* = 0.016).

**TABLE 2:** Baseline characteristics according to lung transplant prioritization.

	Total *N* = 310	Prioritized *N* = 74	Non‐prioritized *N* = 236	*p* value
RECIPIENT CHARACTERISTICS				
Age (years), median (IQR)	42 (27, 55)	33 (24, 50)	45 (29, 56)	**0.007**
Sex, female, *n* (%)	178 (57)	40 (54)	138 (58)	0.502
Primary lung disease, *n* (%)				**< 0.001**
Vascular	16 (5)	9 (12)	7 (3)	
Obstructive	51 (17)	4 (5)	47 (20)	
Restrictive	123 (40)	37 (50)	86 (37)	
Suppurative	119 (39)	24 (32)	95 (40)	
Measured TLC (L), median (IQR)	4.66 (3.31, 6.07)	3.56 (3.03, 4.76)	4.94 (3.56, 6.31)	**< 0.001**
Pulmonary hypertension, *n* (%)	80 (28)	33 (48)	47 (22)	**< 0.001**
Pulmonary artery systolic pressure (mmHg), median (IQR)	51 (45, 70)	56 (50, 82)	49 (45, 61)	**0.016**
Creatinine clearance (mL/min), median (IQR)	98 (80, 119)	99 (83, 120)	98 (80, 119)	0.677
Pre‐transplant clinical status, *n* (%)				**< 0.001**
Non‐hospitalized at the transplant center	221 (74)	5 (7)	216 (95)	
Hospitalized, non‐intensive care unit	52 (17)	44 (60)	8 (4)	
Intensive Care Unit hospitalization	27 (9)	24 (33)	3 (1)	
Time on waiting list (days), median (IQR)	502 (215, 798)	311 (98, 548)	590 (289, 862)	**< 0.001**

DONOR CHARACTERISTICS				
Age (years), median (IQR)	28 (20, 40)	26 (20, 39)	29 (20, 41)	0.735
Sex, female, *n* (%)	190 (61)	48 (65)	142 (60)	0.469
Smoking history, yes, *n* (%)	32 (11)	9 (13)	9 (13)	0.540
Vasoactive drug support, yes, *n* (%)	285 (93)	69 (93)	216 (92)	0.790
Duration of mechanical ventilation (days), median (IQR)	3.00 (2.00, 5.00)	3.00 (2.00, 5.00)	3.00 (2.00, 5.00)	0.530
PaO_2_/FiO_2_ ratio, median (IQR)	409 (356, 452)	398 (349, 461)	411 (362, 450)	0.984
Time from brain death to procurement (hours), median (IQR)	39 (29, 49)	39 (29, 49)	39 (30, 50)	0.954

PERIOPERATIVE CHARACTERISTICS				
Type of lung transplantation, *n* (%)				**0.039**
Unilateral	60 (20)	8 (11)	52 (22)	
Bilateral	247 (80)	64 (89)	183 (78)	
First lung ischemic time (minutes), median (IQR)	290 (250, 325)	285 (250, 320)	290 (250, 330)	0.729
Any ECLS use during transplantation, yes, *n* (%)	89 (29)	49 (66)	40 (17)	**< 0.001**
ECMO use during transplantation, yes, *n* (%)	63 (20)	43 (58)	20 (9)	**< 0.001**
Cell saver–recovered volume (mL), median (IQR)	410 (290, 706)	540 (300, 986)	390 (273, 603)	**0.014**
Colloid volume (mL), median (IQR)	2.00 (1.00, 3.00)	3.00 (2.00, 4.00)	2.00 (1.00, 2.00)	**< 0.001**
Total fluid balance (mL), median (IQR)	2,100 (1,180, 3,105)	2,150 (1,164, 3,300)	2,100 (1,180, 3,100)	0.336
Packed red blood cell transfusion, yes, *n* (%)	146 (54)	58 (81)	88 (44)	**< 0.001**

POSTOPERATIVE OUTCOMES				
Post‐transplant ECMO requirement, yes, *n* (%)	42 (15)	25 (36)	17 (8)	**< 0.001**
PGD grade 3 at 0 h, *n* (%)	72 (23)	25 (34)	47 (20)	**0.012**
PGD grade 3 at 72 h, *n* (%)	33 (11)	16 (24)	17 (7)	**< 0.001**
Tracheostomy, yes, *n* (%)	41 (18)	20 (32)	21 (13)	**0.001**
Pleural drainage > 14 days, yes, *n* (%)	118 (47)	42 (64)	76 (41)	**0.005**
Dialysis requirement, yes, *n* (%)	56 (22)	22 (32)	34 (18)	**0.019**
ICU length of stay (days), mean ± SD	13.4 ± 16.5	18.6 ± 17.6	11.8 ± 15.8	**< 0.001**
Total hospital length of stay (days), mean ± SD	32.8 ± 25.8	38.2 ± 31.5	31.1 ± 23.6	**< 0.001**

Abbreviations: ECLS, Extracorporeal Life Support; ECMO, Extracorporeal Membrane Oxygenation; ICU, Intensive Care Unit; IQR, Interquartile Range; PGD, Primary Graft Dysfunction; SD, Standard Deviation; TLC, Total Lung Capacity;

As expected, prioritized recipients had shorter waiting times (351.5 vs 639 days; *p* < 0.001) and were more frequently hospitalized at transplantation (60% vs 4%; *p* < 0.001), including ICU admission (33% vs 1%; *p* < 0.001). Pre‐transplant ECMO support was also more common among prioritized recipients (58% vs 9%; *p* < 0.001).

### 3.2 Factors Associated With Prioritization

Univariate analyses are shown in Table 3. Younger age (OR 0.98 per year; 95% CI 0.96–0.99; *p* = 0.007) and pulmonary hypertension (OR 3.34; 95% CI 1.88–5.93; *p* < 0.001) were associated with prioritization. Indicators of advanced clinical deterioration showed the strongest associations, including pre‐transplant hospitalization (OR 238; 95% CI 81.1–853; *p* < 0.001) and ICU admission (OR 346; 95% CI 89.5–1873; *p* < 0.001).

**TABLE 3:** Univariable analysis of factors associated with prioritization.

Panel A—Pre‐ and Intraoperative Factors Associated with Prioritization^a^			
Variables	OR	95% CI	*p* value
RECIPIENT‐RELATED			
Age	0.98	0.96–0.99	**0.007**
Pulmonary hypertension	3.34	1.88–5.93	**< 0.001**
Measured TLC	0.73	0.60–0.88	**0.001**
Predicted TLC difference donor/recipient	1.30	1.03–1.67	**0.032**
Donor–recipient blood group mismatch	5.09	2.48–10.6	**< 0.001**
Pre‐transplant hospitalization	238	81.1–853	**< 0.001**
ICU hospitalization	346	89,5–1873	**< 0.001**

PERIOPERATIVE‐RELATED			
Type of transplant, *Unilateral*	0.44	0.19–0.93	**0.043**
Any ECLS use during transplantation	9.60	5.39–17.6	**< 0.001**
ECMO use during transplantation	14.8	7.86–29.0	**< 0.001**
Cell saver–recovered volume	1.00	1.00–1.00	**0.003**
Colloid requirement	1.00	1.00–1.00	**< 0.001**
Packed red blood cell transfusion	5.23	2.80–10.3	**< 0.001**

Panel B—Post‐transplant outcomes in prioritized vs non‐prioritized recipients^b^			
Variables	OR	95% CI	*p* value
Post‐transplant ECMO requirement	6.24	3.14–12.7	**< 0.001**
Tracheostomy	3.12	1.55–6.33	**< 0.001**
Prolonged pleural drainage, *> 2 weeks*	2.21	1.30–3.83	**0.004**
Dialysis requirement	2.11	1.12–3.95	**0.020**
ICU length of stay	1.02	1.01–1.04	**0.004**
Total hospital length of stay	1.01	1.00–1.02	**0.046**
PGD grade 3:			
—0 h	2.08	1.16–3.71	**0.013**
—24 h	4.82	2.36–9.95	**< 0.001**
—48 h	4.26	2.03–8.98	**< 0.001**
—72 h	3.80	1.79–8.06	**< 0.001**

Abbreviations: ECLS, extracorporeal life support; ECMO, extracorporeal membrane oxygenation; ICU, intensive care unit; PGD, Primary Graft Dysfunction; TLC, Total Lung Capacity.

^a^ *Panel A* includes pre‐, donor‐, and intraoperative variables explored as potential correlates with prioritization using univariable logistic regression (OR, 95% CI).

^b^ *Panel B* summarizes postoperative outcomes by prioritization and is presented descriptively; these variables are not interpreted as predictors because of the temporal sequence.

Perioperative factors associated with prioritization included extracorporeal lung support (OR 9.60; 95% CI 5.39–17.6; *p* < 0.001), intraoperative ECMO use (OR 14.8; 95% CI 7.86–29.0; *p* < 0.001), and donor–recipient blood group mismatch (OR 5.09; 95% CI 2.48–10.6; *p* < 0.001). Prioritized recipients also required more packed red blood cell transfusions (OR 5.23; 95% CI 2.80–10.3; *p* < 0.001).

### 3.3 Incidence of Primary Graft Dysfunction

The incidence of PGD grade 3 was consistently higher among prioritized recipients at all time points (Table 3). Immediately after transplantation, PGD grade 3 occurred in 34% of prioritized recipients versus 20% of non‐prioritized recipients (OR 2.08; 95% CI 1.16–3.71; *p* = 0.013). Although this incidence was higher in the prioritized group, the proportion observed among non‐prioritized recipients was also clinically relevant. Among the 72 recipients with PGD grade 3 at 0 h, 25 were prioritized, and 47 were non‐prioritized (Figure 1).

**FIGURE 1:**
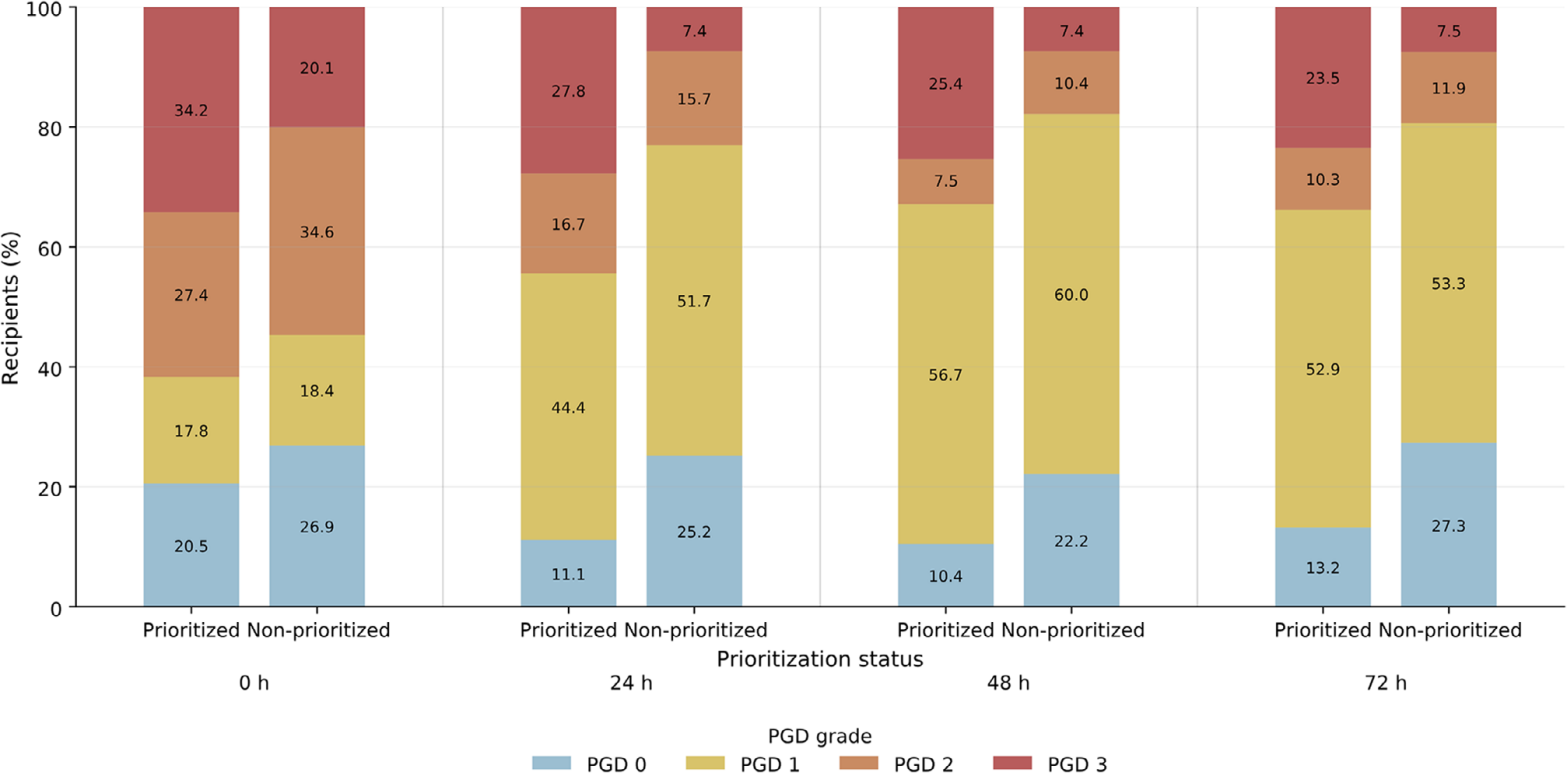
Distribution of primary graft dysfunction grades Over time according to prioritization status. One hundred percent stacked bars show the distribution of primary graft dysfunction (PGD) grades 0–3 Among prioritized and non‐prioritized lung transplant recipients at 0, 24, 48, and 72 h After transplantation. Values Within the bars indicate the percentage of recipients in each PGD category. Percentages were calculated Among recipients With an available PGD assessment at the corresponding time point. PGD grading was performed according to the International Society for Heart and Lung Transplantation criteria.

Restrictive lung disease was the most frequent underlying diagnosis in both groups, accounting for 11/25 cases (44.0%) among prioritized recipients and 22/47 cases (46.8%) among non‐prioritized recipients. Suppurative disease accounted for 6/25 (24.0%) and 16/47 (34.0%) cases, vascular disease for 6/25 (24.0%) and 6/47 (12.8%) cases, and obstructive disease for 2/25 (8.0%) and 3/47 (6.4%) cases, respectively. Pulmonary artery systolic pressure was markedly elevated among recipients with PGD grade 3 at 0 h in both groups, with mean values of 96.5 mmHg among prioritized recipients and 99.5 mmHg among non‐prioritized recipients. The difference in PGD grade 3 incidence increased at 24 h (28% vs 7%; OR 4.82; 95% CI 2.36–9.95; *p* < 0.001) and remained significant at 48 h (25% vs 7%; OR 4.26; 95% CI 2.03–8.98; *p* < 0.001) and 72 h (24% vs 7%; OR 3.80; 95% CI 1.79–8.06; *p* < 0.001).

Interestingly, while prioritized recipients exhibited a higher incidence of severe PGD immediately after transplantation, the prevalence decreased progressively over the first 72 h. In contrast, non‐prioritized recipients who developed PGD grade 3 tended to exhibit persistent dysfunction. Logistic regression analysis showed that both prioritization (*p* < 0.001) and time after transplantation (*p* < 0.001) were associated with severe PGD, whereas the interaction between prioritization and time was not significant (*p* = 0.25) (Figure 2).

**FIGURE 2:**
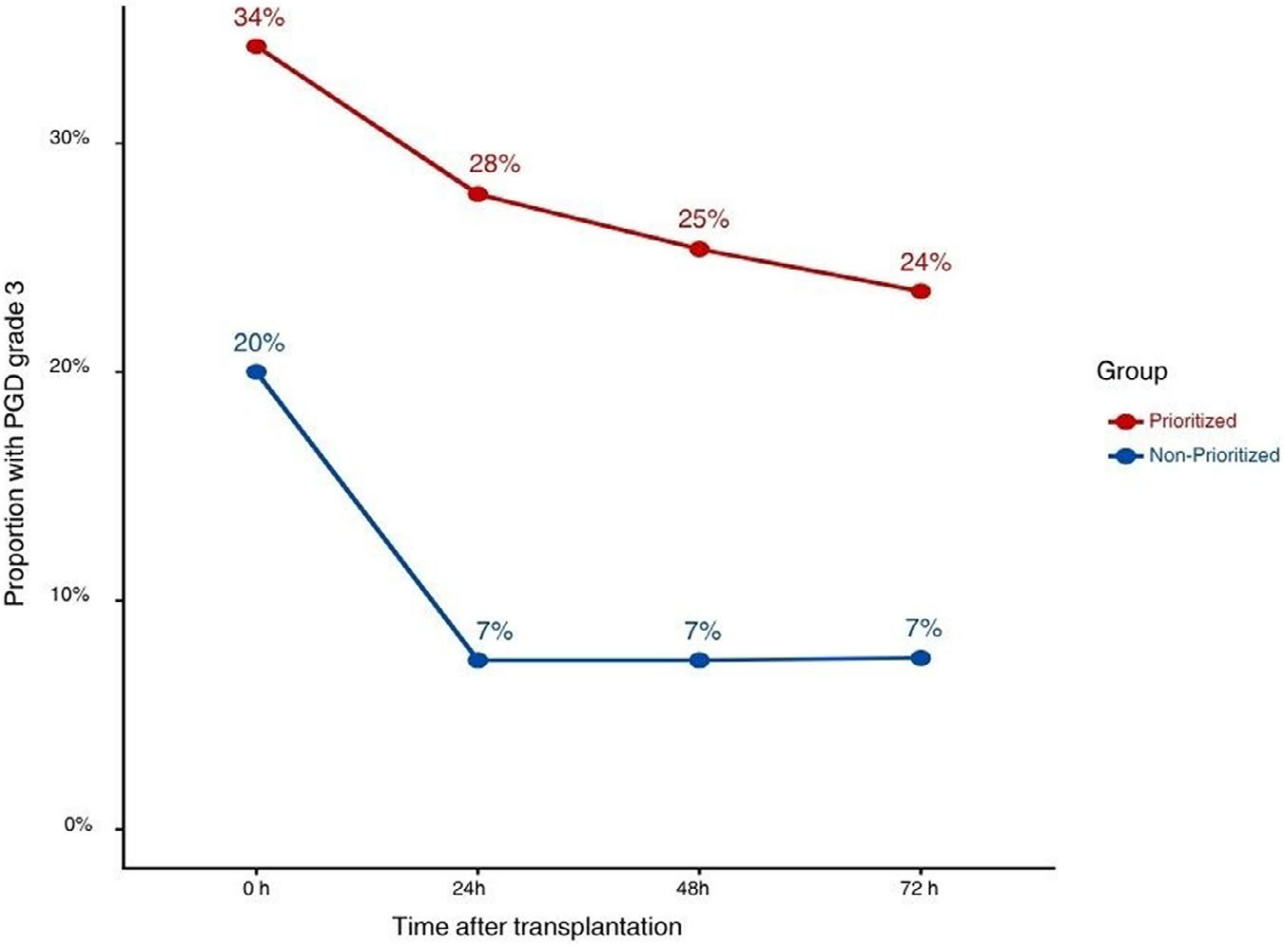
Temporal evolution of severe primary graft dysfunction according to prioritization status. Proportion of recipients with PGD grade 3 at 0, 24, 48, and 72 h after transplantation according to prioritization status. Although the absolute incidence of severe PGD was higher among prioritized recipients, the overall trajectory of PGD severity during the first 72 h did not differ significantly between prioritized and non‐prioritized recipients.

### 3.4 Post‐Transplant Outcomes

Prioritized recipients experienced higher rates of several postoperative complications (Table 3), including post‐transplant ECMO requirement (36% vs 8%; OR 6.24; 95% CI 3.14–12.7; *p* < 0.001), dialysis (32% vs 18%; OR 2.11; 95% CI 1.12–3.95; *p* = 0.020), tracheostomy (32% vs 13%; OR 3.12; 95% CI 1.55–6.33; *p* < 0.001), and prolonged pleural drainage (64% vs 41%; OR 2.21; 95% CI 1.30–3.83; *p* = 0.004).

Prioritized recipients had longer ICU stays than non‐prioritized recipients (18.6 ± 17.6 vs 11.8 ± 15.8 days; *p* < 0.001) and longer overall hospital length of stay (38.2 ± 31.5 vs 31.1 ± 23.6 days; *p* < 0.001).

### 3.5 Factors Associated With Severe PGD at 72 H

After adjustment for recipient age, pulmonary hypertension, ischemic time, and packed red blood cell transfusion, pre‐transplant ECMO support remained independently associated with severe PGD (OR 4.57; 95% CI 1.30–17.38; *p* = 0.020). In contrast, prioritization status was not independently associated with PGD grade 3 after adjustment for these clinical severity markers (OR 1.06; 95% CI 0.31–3.47; *p* = 0.93).

Among recipients with pulmonary hypertension, 20 (25%) developed PGD grade 3 at 72 h without requiring postoperative ECMO. This subgroup represented 60% of all PGD grade 3 cases at 72 h. These findings support the concept that severe pulmonary hypertension may contribute to reperfusion injury and PGD classification even in the absence of postoperative extracorporeal support.

### 3.6 Survival

Survival analysis according to prioritization status is shown in Figure 3. Thirty‐day mortality did not differ between prioritized and non‐prioritized recipients. However, mortality was higher among prioritized recipients at 90 days (32% vs 20%) and at 1 year (54% vs 35%). Kaplan–Meier curves demonstrated early separation during the first year after transplantation, and the overall log‐rank comparison showed worse survival among prioritized recipients.

**FIGURE 3:**
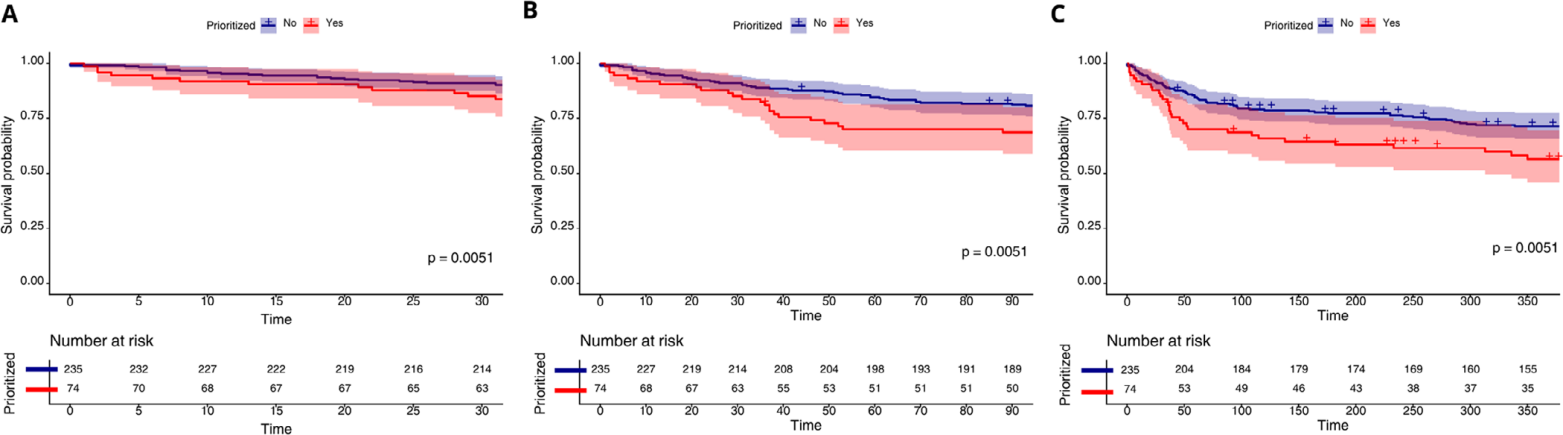
Kaplan–Meier survival curves stratified by prioritization status for lung transplantation. Post‐transplant survival Among prioritized and non‐prioritized lung transplant recipients is shown at 30 days (A), 90 days (B), and 1 year (C) After transplantation. Panels represent the same Kaplan–Meier analysis displayed at different follow‐up horizons. The *p*‐value shown corresponds to the overall log‐rank comparison Between prioritization groups and is therefore identical Across panels. Numbers at risk are displayed Below each panel.

### 3.7 Cox Proportional Hazards Model Analysis

Cox regression analysis (Table S2) identified several factors associated with mortality. Pre‐transplant ICU hospitalization increased mortality risk (HR 2.48; 95% CI 1.17–5.28; *p* = 0.018). Perioperative extracorporeal life support (HR 1.95; 95% CI 1.30–2.92; *p* = 0.001) and cardiopulmonary bypass (HR 2.09; 95% CI 1.29–3.37; *p* = 0.003) were also associated with worse survival. Postoperative complications showed the strongest associations with mortality, particularly post‐transplant ECMO (HR 4.22; 95% CI 2.67–6.65; *p* < 0.001), dialysis (HR 6.17; 95% CI 4.10–9.28; *p* < 0.001), and tracheostomy (HR 5.39; 95% CI 3.32–8.74; *p* < 0.001). Severe PGD at multiple time points, including PGD grade 3 at 72 h, was also strongly associated with increased mortality (HR 2.64; 95% CI 1.65–4.22; *p* < 0.001).

## 4 Discussion

In this cohort of lung transplant recipients, transplantation performed under prioritization was associated with a higher crude incidence of severe PGD and worse early survival. Prioritized recipients exhibited higher rates of PGD grade 3 at all evaluated time points during the first 72 h after transplantation. However, after adjustment for clinical severity markers, prioritization itself was not independently associated with PGD, whereas pre‐transplant ECMO support remained a strong independent predictor of severe graft dysfunction. These findings suggest that the higher incidence of PGD observed among prioritized recipients may be explained by the severity of illness leading to prioritization rather than by prioritization status itself.

Prioritization policies aim to provide timely transplantation to candidates with rapidly deteriorating clinical status or high risk of waitlist mortality. In our cohort, prioritized recipients were younger and exhibited a higher prevalence of pulmonary hypertension, as well as more frequent hospitalization, particularly in the ICU. These findings reflect the greater baseline clinical acuity of prioritized candidates, who often represent critically ill patients requiring urgent transplantation. In many allocation systems, such a high‐priority status is granted to patients requiring advanced support as a bridge to transplantation [3, 12]. These conditions have been consistently associated with increased peri‐transplant risk and worse early outcomes after LTx.

Prioritized recipients demonstrated markedly higher rates of PGD grade 3 immediately after transplantation and throughout the early postoperative period. This observation is biologically plausible, as these patients frequently present with advanced respiratory failure, systemic inflammation, and hemodynamic instability prior to transplantation [2]. Many require prolonged mechanical ventilation and extracorporeal support before transplantation, conditions that may amplify systemic inflammatory responses and predispose the graft to reperfusion injury [13]. These conditions likely reflect increased physiological vulnerability that persists throughout the perioperative period and contributes to the higher incidence of early graft dysfunction.

Although the absolute incidence of severe PGD was higher among prioritized recipients, the temporal trajectory of PGD during the first 72 h did not differ significantly between prioritized and non‐prioritized recipients. Prioritized recipients appeared to demonstrate a progressive reduction of PGD grade 3 over time, whereas severe PGD among non‐prioritized recipients remained relatively stable after the first postoperative day. Although this difference did not reach statistical significance, it suggests that a subset of prioritized recipients may experience partial early recovery from ischemia–reperfusion injury despite presenting with more severe initial dysfunction.

In our multivariable analysis, pre‐transplant ECMO support remained the only independent predictor of PGD grade 3 at 72 h. Patients bridged to transplantation with ECMO represent a particularly vulnerable population [14] that has increased in recent years, particularly where the lung allocation score (LAS) was adopted [15]. Prolonged extracorporeal support may lead to systemic inflammatory activation, endothelial dysfunction, and coagulation abnormalities, increasing susceptibility to ischemia–reperfusion injury following lung implantation [16]. Previous studies have similarly identified pre‐transplant ECMO as an important risk factor for PGD and early post‐transplant complications [9, 13, 17].

Despite the higher incidence of severe PGD and postoperative complications among prioritized recipients, mortality differences did not emerge immediately after transplantation. Thirty‐day mortality was similar between groups, whereas higher mortality among prioritized recipients became evident at 90 days and 1 year. These findings suggest that the excess risk associated with prioritization reflects the cumulative impact of early graft dysfunction, postoperative complications, and the physiological fragility of critically ill recipients. Severe PGD has been strongly associated with both short‐term mortality and long‐term allograft dysfunction [7, 9, 18]. Overall survival in this cohort was lower than that reported in contemporary ISHLT registry data. This difference should be interpreted in light of the study setting and case mix. Our cohort included a substantial proportion of critically ill recipients, patients with pulmonary hypertension, recipients requiring ECMO or other extracorporeal support, and candidates transplanted under urgency‐based prioritization. In addition, differences in donor availability, access to rehabilitation, ICU discharge pathways, and post‐transplant resource organization in a middle‐income healthcare system may contribute to outcome differences compared with large international registry populations. These factors limit direct comparison with LAS‐based systems and reinforce the importance of interpreting our findings within the Brazilian allocation context.

These findings have important implications for the management of transplant candidates and allocation strategies. As advances in extracorporeal support and perioperative care expand transplant eligibility, prioritization may increasingly select recipients with greater baseline risk for early graft injury [2, 19]. Our findings suggest that the elevated PGD incidence in prioritized recipients is largely attributable to pre‐transplant disease severity rather than to prioritization itself. Strategies aimed at mitigating ischemia–reperfusion injury—including optimized donor management, lung preservation techniques, and careful perioperative hemodynamic management—may therefore be particularly important in this high‐risk population [19].

Importantly, the prioritization criteria applied in this study were based on former Brazilian legislation [10] and, therefore, are not directly comparable to the Lung Allocation Score (LAS) used in other countries. Unlike LAS‐based systems, which rely on a continuous score that incorporates predicted waitlist mortality and post‐transplant survival [20], the Brazilian allocation model has historically relied primarily on chronological waiting time, with prioritization granted only in specific clinical circumstances. Candidates with rapid respiratory deterioration—such as refractory respiratory failure or prolonged ECMO support—could receive prioritization after individual evaluation by the State Transplant Center Technical Chamber. In 2025, a new national regulation was implemented [21], introducing revised prioritization criteria based on hierarchical severity levels. These include, in descending order of urgency, acute graft dysfunction, the use of ECLS, the requirement for invasive mechanical ventilation, and finally, waiting time on the transplant list. Table 1 compares the LAS‐based allocation with the Brazilian prioritization system during the study period and the new Brazilian allocation framework established in 2025.

Several limitations should be acknowledged. First, this was a retrospective single‐center analysis, which may limit generalizability to programs with different allocation systems or perioperative practices. Second, although the cohort included all consecutive lung transplant recipients over nine years, the number of prioritized recipients remained relatively limited. Third, unmeasured confounding factors related to donor quality, perioperative management, or inflammatory status may have influenced outcomes. Fourth, ICU length of stay should be interpreted within the context of local institutional practice, as lung transplant recipients are often maintained in the ICU beyond the immediate phase of critical illness for intensive monitoring and complex postoperative care. In addition, detailed data on the duration of mechanical ventilation were not consistently available across the entire study period because of changes in data collection practices; therefore, this variable was not included in the final analysis. Finally, while PGD grade 3 at 72 h was used as a clinically meaningful endpoint, future studies incorporating biomarkers and mechanistic data may further clarify the pathways linking recipient severity, extracorporeal support, and early graft injury.

## 5 Conclusion

Transplantation performed under prioritization had a higher crude rate of severe PGD and worse early survival after LTx. However, after adjustment for clinical severity markers, pre‐transplant ECMO support—not prioritization status itself—was the primary independent predictor of severe PGD. These findings highlight the central role of recipient critical illness in determining early graft outcomes and underscore the importance of optimizing perioperative management strategies for prioritized transplant candidates.

## Author Contributions

**FPR**: conceptualization; methodology; project administration; visualization; writing — original draft; writing — review & editing. **AMT**: writing — original draft; writing — review & editing. **SLS**: methodology; writing — original draft; writing — review & editing. **FFAV**: writing — original draft; writing — review & editing. **VAC**: Writing—original draft; Writing—review & editing. **LGA**: Writing—original draft; Writing—review & editing. **SVC**: Writing—original draft; Writing—review & editing. **LMF**: Writing—original draft; Writing—review & editing. **PMFP**: Writing—original draft; Writing—review & editing.

## Funding

This research and its authors received no specific grant From any funding agency in the public, commercial, or not‐for‐profit sectors.

## Ethics Statement

The study was approved by the local Institutional Ethics Committee (CAAE: 39990120.8.0000.0068), and written informed consent was obtained from all participants or parents/guardians if participants were underage.

## Conflicts of Interest

The authors certify that they have no affiliations with or involvement in any organization or entity with any financial interest in the subject matter or materials discussed in this manuscript.

## Declaration of Generative AI in Scientific Writing

During the preparation of this work, the authors used ChatGPT 5.1, OpenAI, in order to improve readability and language. After using this tool, the authors reviewed and edited the content as needed and take full responsibility for the content of the publication

## Statements and Declarations

The Article Processing Charge for the publication of this research was funded by the Brazilian Federal Agency for Support and Evaluation of Graduate Education — CAPES (ROR identifier: 00×0ma614). For open access purposes, the authors have applied a Creative Commons CC BY license to any accepted version of the article.

## Supporting information




**Supporting File 1:** ctr70711‐sup‐0001‐SupMat.pdf.

## Data Availability

All data is available from the corresponding author upon request.
