# Supplementary material for: Prioritization and Severe Primary Graft Dysfunction after Lung Transplantation
Source: Clin Transplant. 2026 Oct 14;40(10):e70711. doi: 10.1111/ctr.70711 (PMC13664657; doi:10.1111/ctr.70711)
Supplement: Supplementary file 1 [file CTR-40-e70711-s001.pdf]

# SUPPLEMENTARY INFORMATION

**Supplementary Table 1 - ISHLT definition and severity grading of primary graft dysfunction: 2016 consensus published in 2017**

| Grade | Pulmonary edema on chest X-ray | $Pa_{O_2}/F_{IO_2}$ ratio |
|---|---|---|
| **PGD grade 0** | No | Any |
| **PGD grade 1** | Yes | >300 |
| **PGD grade 2** | Yes | 200 to 300 |
| **PGD grade 3** | Yes | < 200 |

**Grade severity notes:** Patients with no evidence of pulmonary edema on chest X-ray are considered grade 0. Absence of invasive mechanical ventilation should be graded according to the Pao2/Fio2 ratio, using methods similar to those receiving mechanical ventilation.

If Pao2 is not available for calculation of a Pao2/Fio2 ratio, then an oxygen saturation/Fio2 ratio should be calculated and the 200 and 300 PGD grading cutoffs should be adjusted to 235 and 315. Use of nitric oxide, aerosolized epoprostenol, or other pharmacologic agents that may improve oxygenation should not change grading methods. Use of extracorporeal lung support (ECLS) with bilateral pulmonary edema on chest X-ray image should be graded as grade 3 and ECLS use should be explicitly recorded. The use of ECLS for non-hypoxic indications without pulmonary edema on chest X-ray imaging should be considered un-gradable and explicitly recorded separately.

**Time window notes:** PGD is graded at 4 time-points, every 24 hours, over the first 72 hours after transplantation (T0, T24, T48, and T72 hours). Time starts at reperfusion of second lung. T0, T24, T48, and T72 have time windows ± 6 hours. If multiple blood gas values are available, the worst Pao2/Fio2 ratio on a given calendar day should be used.

**Other notes:** Pao2/Fio2 should ideally be measured on positive end-expiratory pressure of 5 cm H2O at Fio2 of 1.0 while patients are on mechanical ventilation; however, grading should not be altered for other settings. For altitudes higher than 1,000 m, use of an appropriate correction factor is recommended. For comparative purposes, single lung transplantation is recommended to be reported separately from bilateral lung transplant without change in grading methodology.

$F_{IO_2}$, *fraction of inspired oxygen*; $Pa_{O_2}$, *partial pressure of arterial oxygen*; PGD, *primary graft dysfunction*.

*Adapted from Snell et al.*[1]

**Supplementary Table 2. Cox proportional hazards analyses of recipient, perioperative, and postoperative factors associated with mortality**

| Variable | HR | 95% CI | p-value |
|---|---|---|---|
| **Recipients related variables** | | | |
| Six-minute walk test performance > 450 meters | 1.50 | 1.05-2.13 | **0.025** |
| Pre-transplant Intensive Care Unit hospitalization | 2.48 | 1.17-5.28 | **0.018** |
| **Perioperative-related variables** | | | |
| ECLS use during transplantation, yes | 1.95 | 1.30-2.92 | **0.001** |
| Cardiopulmonary bypass use, yes | 2.09 | 1.29-3.37 | **0.003** |
| ECMO use, yes | 1.60 | 1.00-2.55 | **0.048** |
| Cell saver–recovered volume | 1.00 | 1.00-1.00 | **0.050** |
| **Post-operative outcomes** | | | |
| Time in mechanical ventilation | 1.00 | 1.00-1.00 | **<0.001** |
| Packed red blood cell transfusion, yes | 1.51 | 1.04-2.19 | **0.031** |
| Post-transplant ECMO requirement, yes | 4.22 | 2.67-6.65 | **<0.001** |
| PGD grade 3 at 0h, yes | 2.08 | 1.44-3.00 | **<0.001** |
| PGD grade 3 at 24h, yes | 2.89 | 1.85-4.52 | **<0.001** |
| PGD grade 3 at 48h, yes | 2.78 | 1.73-4.46 | **<0.001** |
| PGD grade 3 at 72h, yes | 2.64 | 1.65-4.22 | **<0.001** |
| Tracheostomy, yes | 5.39 | 3.32-8.74 | **<0.001** |
| Pleural drainage > 14 days | 1.75 | 1.19-2.56 | **0.004** |
| Dialysis requirement, yes | 6.17 | 4.10-9.28 | **<0.001** |
| ICU length of stay | 1.02 | 1.02-1.03 | **<0.001** |

*ECLS*, extracorporeal life support; *ECMO*, extracorporeal membrane oxygenation; *ICU*, intensive care unit.

## SUPPLEMENTARY REFERENCES


1. Snell GI, Yusen RD, Weill D, et al. Report of the ISHLT Working Group on Primary Lung Graft Dysfunction, part I: definition and grading. *J Heart Lung Transplant.* 2017;36(10):1097-1103. doi:10.1016/j.healun.2017.07.021
